# Bioengineering the Cardiac Conduction System: Advances in Cellular, Gene, and Tissue Engineering for Heart Rhythm Regeneration

**DOI:** 10.3389/fbioe.2021.673477

**Published:** 2021-08-02

**Authors:** Nataliia Naumova, Laura Iop

**Affiliations:** Department of Cardiac Thoracic Vascular Sciences and Public Health, University of Padua, Padua, Italy

**Keywords:** biological pacemaker, cardiac conduction system, cardiac diseases, bioengineering, gene engineering, tissue engineering

## Abstract

Heart rhythm disturbances caused by different etiologies may affect pediatric and adult patients with life-threatening consequences. When pharmacological therapy is ineffective in treating the disturbances, the implantation of electronic devices to control and/or restore normal heart pacing is a unique clinical management option. Although these artificial devices are life-saving, they display many limitations; not least, they do not have any capability to adapt to somatic growth or respond to neuroautonomic physiological changes. A biological pacemaker could offer a new clinical solution for restoring heart rhythms in the conditions of disorder in the cardiac conduction system. Several experimental approaches, such as cell-based, gene-based approaches, and the combination of both, for the generation of biological pacemakers are currently established and widely studied. Pacemaker bioengineering is also emerging as a technology to regenerate nodal tissues. This review analyzes and summarizes the strategies applied so far for the development of biological pacemakers, and discusses current translational challenges toward the first-in-human clinical application.

## Introduction

Cardiac and cardiovascular diseases are globally increasing due to the aging of populations. The total number of electronic cardiac pacemaker implantations has risen accordingly (Mensah et al., 2019; Bai et al., 2020; Peters et al., 2020; Virani et al., 2020). However, even with continuous technological improvements, current-generation electronic pacemakers still have significant limitations and complications regarding the clinical application. Electronic pacemaker implantations are accompanied with numerous challenges, such as the risk of various infections, a relatively short battery life, lead failure/repositioning, pacemaker material allergy, electronic interference, an occasional component failure, vascular, and other complications (Nishii, 2016). Several cardiac conditions require additional non-device approaches as in the case of a congenital heart block, which cannot be treated using electronic pacemakers, or in the case of a contraindication to reimplantation before effective antibiosis is established. A congenital heart block and other congenital cardiac conditions could result in fetal death or stillbirth and, in most cases, require *in utero* pacing (Gutiérrez et al., 1989; DeSimone and Sohail, 2018; Manolis et al., 2020).

Biological pacemakers could be, therefore, a new promising therapeutic alternative to current electronic devices, being the advanced, effective biotechnology to counter these challenges. The concept of biological pacemakers is based on bioengineering and biotechnology approaches for the production and implantation of different pacemaker cellular components for the electrical pacing of heart. As of date, numerous studies are conducted, and methodologies are proposed to create clinically relevant biological pacemakers as an alternative to the artificial cardiac devices.

## Anatomy, Biology, and Physiology of the Cardiac Conduction System

The mammalian cardiac conduction system can be figured as an electrical path able to generate the impulse and transfer it across the heart, where it triggers the electromechanical force at the base of the pump function. In this electrical path, several stations with a precise functional hierarchy are present: firstly, the sinoatrial node (SAN); then, the atrioventricular node (AVN), the His bundle, the left and right bundle branches; and, finally, the Purkinje fibers (Park and Fishman, 2011; Persson and Persson, 2012).

### The Sinoatrial Node

SAN is a natural pacemaker of the heart, i.e., the specialized myocardial tissue responsible for our 2-billion heartbeats in the lifespan. Described for the first time by Keith and Flack in 1907 as a “wonderful structure” Keith and Flack (1907), the SAN is the heart conduction system's primary station.

In almost all mammals, SAN is localized sub-epicardially in the *sulcus terminalis*, namely the terminal groove, in the junctional region between the right atrium and superior vena cava (Liu et al., 2007). In most cases, it displays a tadpole shape with a length varying from 1.5 mm in mouse to 15 mm in humans (James, 1961; Liu et al., 2007), but large variability in size and localization has been observed in intraspecies and interspecies anatomical comparisons (James, 1977).

Being already distinguishable from 6 to 8 weeks of embryonic development, this specialized tissue has a particular composition in cells and extracellular matrix elements, rendering it very particular with respect to the working myocardium and other adjacent structures. An intricate network of collagens surrounds, in humans, the cells responsible for the generation of the impulse, the so-called pacemaker cells (PCs), and other cytotypes, i.e., the transitional cells. Each PC displays a clear cytoplasmic zone around a large, centrally located nucleus with little glycogen amount and randomly oriented myofibrils and small mitochondria. Proximity to and arrangement in clusters or grapes are mainly observed among PCs, but the interconnection is poor with the absence of intercalated disks and tight junctions (James, 1977). During SAN depolarization, the spontaneous release of Ca^2+^ from the sarcoplasmic reticulum through the ryanodine receptor 2 (RYR2) activates the five distinct ionic currents of PCs, i.e., (1) the voltage-dependent outward current I_k_, generated by ERG channels, (2) the inward current carried by Na^+^ and K^+^ ions, the so-called funny current I_f_, dependent on HCN1/4 channels, (3) the L-type Ca^2+^ current, i.e., I_Ca,L_, generated by Cav1.2/1.3; (4) the T-type Ca^2+^ current, i.e., I_Ca,T_ mediated by Cav3.1/3.2 channels, and, finally, (5) the inward Na^+^-Ca^2+^ exchange current, i.e., I_NaCa_ due to the NCX1 channels. All these membrane ion channels concur to the physiological automaticity of SAN, but a long debate is still going on the predominant one in controlling the spontaneous diastolic depolarization (DD) (Lakatta and DiFrancesco, 2009; DiFrancesco, 2020). I_f_ current is largely considered the electrophysiological hallmark of SAN. It was discovered as an inward current in 1979 by the electrophysiological analysis of the cell preparation of a rabbit sinoatrial node. Indeed, an inward current was previously observed in the hearts of mammals and amphibians (Noma and Irisawa, 1976; Brown et al., 1977): however, its relevance was not immediately recognized, nor the contributing ion channel(s) were distinguished. The unusual behavior and features of the just identified current induced its discoverers Brown and colleagues to describe it as “funny.” It was, in fact, unprecedented that an ionic current was revealed to be activated by hyperpolarization at a very low threshold, works at the voltage range that includes the DD voltage diapason (from −40/50 to −100 mV), and display kinetics characterized by reverse at about −10/−20 mV thanks to the Na^+^/K^+^ channel permeability (Brown et al., 1979). Not only I_f_ was demonstrated to possess these unique properties, but also showed responsiveness to adrenergic and muscarinic stimulations. All the fundamental requisites for DD generation and heart rate modulation are indicative of the pacemaking ability of I_f_ current. Lately, another feature was found to render I_f_ unique, namely its physiological retrieval in almost no other body tissues than SAN (Liu et al., 2007). Another inward current gained interest as a possible “intracellular Ca^2+^ clock,” i.e., I_NaCa_: Ca^2+^ cycling at the sarcolemmal membrane is particularly relevant for normal pacemaker automaticity and, hence, it could represent another mechanism of pacemaking (Maltsev and Lakatta, 2008). During DD, in fact, NCX channels become activated by the spontaneous, local Ca^2+^ releases after the opening of RYR2. As a consequence, the depolarization of the membrane potential is increased until the threshold for the next action potential is reached. Uncertainty still remains about the relative roles of I_f_ (membrane voltage clock) and I_NaCa_ (intracellular Ca^2+^ clock) in the normal total pacemaker clock (Lakatta and DiFrancesco, 2009). Conversely, the conclusive phase of the action potential in PCs has been more defined, with Ca^2+^ re-uptaking in the sarcoplasmic reticulum *via* Ca^2+^ ATPase SERCA2 (Irisawa et al., 1993; Mangoni et al., 2006; Maltsev and Lakatta, 2008; Chandler et al., 2009).

Around PC grape-like clusters, transitional cells distribute in the outer half part of SAN. Despite a similar elongated aspect to working myocardial cells, transitional elements are smaller in size, very interweaved, abundant in glycogen, less rich in myofibrils and mitochondria, distributed in an arranged fashion. Transitional cells are much higher in number than PCs and are responsible for transmitting the sinus impulse to the ordinary myocardium. These SAN cells have an embryonic origin in the *sinus venosus* as other cell types composing the node, i.e., fibroblasts and nerve cells.

An important component of SAN is its main artery, prevalently originating from the right coronary artery as its first branch. The SAN artery crosses the node centrally and is widely interconnected with the right and circumflex coronary tree, thus supplying blood to the atrium and the same node. A copious net of capillaries, arterioles, and venules supports the vascularization of the whole SAN.

Due to its proximity and arterial connection to the aorta, SAN also exerts a sensor role for monitoring central aortic pressure and pulse, a servomechanism stabilizing pulse and impulse. Moreover, adrenergic and cholinergic nerves operate as further functional stabilizers in the beat-to-beat frame and in the longest time. In particular, the balanced autonomic innervation contributes to the stable performance of SAN in its postnatal maturity (James, 1970).

### The Atrioventricular Node

AVN lies at the conjunction of the right atrium and the right ventricle. For this purpose, Tawara (1906) described it as the sole electrical connection between the upper and lower chambers of heart as he first observed in several mammalian species. This anatomical hypothesis was confirmed after 1 year by Keith and Flack (1907) and further elaborated 7 years later by Kent (1913) as a multiple, muscular atrioventricular path. Knowledge about AVN was progressively re-evaluated with an increase in interest not only by anatomists and histologists but also by molecular/clinical cardiologists and electrophysiologists (Anderson and Siew, 2002).

This second station of the conduction system appears as a small spindle-shaped structure localized in humans at the apex of the Koch triangle, an endocardial region ideally defined by the coronary sinus orifice, the Todaro tendon, and the septal leaflet of the tricuspid valve. AVN receives blood supply *via* the right coronary artery in the right heart dominance case (Kurian et al., 2010; Anderson et al., 2020). Besides a compact nodal area, a transitional region is present. Two electrophysiologically distinct conduction pathways connect SAN to AVN. The pathways were described first in 1971 (Spach et al., 1971) as the “fast AVN” and the “slow AVN” ones as they are the fastest and slowest pathways for the action potential *via* AVN (Dobrzynski et al., 2013). Therefore, AVN is characterized by a “dual-pathway electrophysiology”: the route *via* the interatrial septum connects to the fast (normal) pathway, and the route *via* the terminal crest connects to the slow pathway (Meijler and Janse, 1988; Dobrzynski et al., 2013).

The inferior extension of AVN is much debated, which recently has been better characterized in human hearts (Anderson et al., 2020; Cabrera et al., 2020). In particular, wide variability was described in terms of dimension and penetration degree for the AVN conduction tracts entering the myocardium as well as for the subsequent part of the conduction system, i.e., the bundle of His (further details in the next subchapter). Intrahuman variability has been observed for many AVN components, such as the nodal region morphology and the connections of atrial myocardium (Anderson et al., 2020; Sternick and Sánchez-Quintana, 2021).

AVN generates cardiac automaticity only as a subsidiary to SAN and with different characteristics from the first conduction system station. AVN shows a filtering activity protecting the ventricles from supraventricular tachycardia. Its conduction velocity is slower (AVN delay); thus, the potential action transmission is highly controlled, and the atrial and ventricular excitation–contraction cycle is very coordinated also in the case of atrial fibrillation or tachycardia. AVN-generated automaticity is only subordinate to SAN and might impose only with the dysfunctional pacemaking of the latter (Meijler and Janse, 1988). It has been reported that a minimum of three different functional regions and five different cell types constitute AVN. From AVN cell types, cell junctions with ventricular bundle branch and His cells are established. Like SAN PCs, midnodal cells are very packed in the so-called Tawara's “Knote” or N region, display scarce myofibrils, and rarely establish junctional relationships (Meijler and Janse, 1988). They express HCN4 (Huang et al., 2013; Xia et al., 2020) and depend on voltage-gated L-type Cav1.3 and T-type Cav3.1 Ca^2+^ channels, too (Baudot et al., 2020), through which pacemaking can be controlled when required. Transitional cells derive their name from the AVN midnodal region transition toward the atrial myocardium. In the rabbit heart, where they have been deeply studied, three transitional cell types have been distinguished mainly depending on their location (Anderson et al., 1974). Lower nodal cells are arranged in a bundle parallel to AVN and display a smaller size than atrial cells (Meijler and Janse, 1988). The NH region identifies the cells that connect with the His bundle and are thought to be the sites of AVN automaticity. The AVN nodal delay depends on the transmission through these multiple bundles and a peculiar arrangement of connexins in the nodal cells. In addition, the diameter of these latter concurs in the transmission delay as well as the possible presence of passive electrical properties (Pollack, 1976; Meijler and Janse, 1988; LeBlanc and Dubé, 1993; Choi and Salama, 1998). The typical connexins of the physiologic human myocardium, namely Cx43, 45, 40, and 31.9, show a differential distribution in AVN by combining in specific ratios and in a reduced number of gap junctions. For example, Cx43, being very abundant in the working myocardium, is only expressed in the AVN penetrating bundle, whereas Cx45 is present in both the compact node and inferior extension (Dobrzynski et al., 2013).

The AVN delay might be related to the relative paucity of connexins, especially Cx43, as well as to the reduced number of Na_v_1.5 channels and hence, the dependence on a slow inward ion current, as firstly hypothesized by Rougier et al. (1969) in the frog atrium and by Zipes and Mendez (1973) in the isolated rabbit heart.

### The His Bundle, Bundle Branches, and Purkinje Fibers

The discovery of the His bundle by its homonymous researcher dates to almost 15 years before identifying SAN and AVN. It was only at the beginning of the 1970s that James and Sherf (1971) differentiated histologically the regions belonging to AVN and the His bundle.

Anatomically, the His bundle appears as a group of fibers with surrounding electrical isolation provided by the central fibrous body. This fascicle exits AVN, and mainly enters the membranous septum at the apex of the Koch triangle to continue afterward into the left and right bundle branches. Through its triple ramification, the thicker left bundle branch is responsible for the excitation of the mid-septal area and the regions of the anterolateral and the posteromedial papillary muscles. The right bundle branch travels toward the moderator band and the anterolateral papillary muscle. Lastly, they both culminate in the sub-endocardium, respectively, in the left and the right ventricles, by connecting with Purkinje fibers that contribute to the transmission of the electrical signal to the endocardial ventricular regions (Padala et al., 2021).

Compared to the surrounding cells, Purkinje cells are histologically distinguishable thanks to their clear cytoplasm, as observed first by Jan Purkinje in 1839 (Cavero et al., 2017). Unlike the cells composing the upper portions of the conduction system, Purkinje fibers display no electrical isolation from the rest of the cardiac tissues although they equally propagate the electrical impulse and show pacing and triggered activity but to a minimal extent. Interestingly, the type and distribution of connexins, namely Cx40 and 43, are peculiar to Purkinje cells with respect to the cells constituting the His bundle. Moving from the His bundle toward Purkinje fibers, the expression of Cx40 increases. Cx43 cellular localization becomes membranous and copious only in Purkinje cells that interact among themselves and not with other sub-endocardial cells (Dun and Boyden, 2008). Regarding action potentials, the duration is longer than in ventricular cells due to prolonged cell repolarization mainly dependent on I_KAch_, but depolarization is short thanks to very rapid, I_Na_-related maximal upstroke velocity (Gintant et al., 1984; Yang et al., 1996). Other differences can be identified in the electrophysiological behavior of Purkinje cells, as reviewed by Dun and Boyden (2008).

## Approaches for the Engineering of Biological Pacemakers

With such a sophisticated composition and functionality, both congenital and acquired cardiac rhythm disturbances may originate from a heterogeneous spectrum of pathophysiological mechanisms (Persson and Persson, 2012). Therefore, effective anti-arrhythmic therapeutic approaches are more and more in demand for both pediatric and adult patients.

When pharmacological treatment is inefficacious to treat arrhythmias, artificial devices must be implanted to control pacing and prevent lethal consequences. These artificial treatments are lifesaving but show many shortcomings associated with a possible generator malfunction, the lack of autonomic responsiveness, a short battery lifespan, undesirable interactions with strong magnetic fields, device-related infections, etc. (Cingolani et al., 2018; Co et al., 2021; Joury et al., 2021). Additional challenges are related to artificial device implantation in children during the existence of the conditions of rapid growth, small body size, and/or anatomical variations associated with congenital heart defects (Cingolani et al., 2018; Taleski and Zafirovska, 2021). Other limitations are associated to the cardiac patients' required continuous monitoring and implementation of early interventions (Joury et al., 2021; Taleski and Zafirovska, 2021).

Various strategies have been progressively designed and improved to restore physiological cardiac pacing through natural solutions. The first attempt to restore physiological pacing was realized in several mammal species at the end of the 1920s by transplanting autologous, allogeneic, or xenogeneic conduction system tissue, namely a pedicle of the right atrium containing SAN, in the ventricular myocardium. The intervention was disclosed generally successful in case of autologous transplantation with the established rhythm between 6 h and 5 days from heterotopic grafting. Although effective for the first hours, allogeneic and xenogeneic grafts became progressively dysfunctional and underwent fibrosis (Rylant, 1927). About 40 years later, several preclinical studies were proposed to re-establish the electrical conduction between the right auricle and ventricle in a canine model of normal heart or complete block. By applying the same pedicle technique, autologous SAN tissues demonstrated to pace only after 2 months from the implantation in the normal heart (Ernst and Paulson, 1962). After a stable, complete heart block, the implanted autologous SANs established no influence on the recipient's preexistent idioventricular rhythm (Starzl et al., 1963). Uncertainties on the validity of the surgical technique applied and other technological difficulties related to the implantation and monitoring of the conduction tissue (e.g., pacemaker malfunctions) were also evident with further investigation (Morishita et al., 1981). Although not completely clear in the eyes of the scientists who tried it first, this initial approach could not be successful for many reasons, starting with a possible immune response to the allogeneic/xenogeneic tissue (foreign body reaction) up to the difficulties to achieve electrical integration and coupling with recipient's myocardium. With the current knowledge, one of the main causes of this unsuccess—besides immunoreaction—was the phenomenon of core necrosis (Rouwkema et al., 2009), which is a consequence of the blood supply insufficiency and a sign of failed tissue integration, very well-known to bioengineers.

**Figure 1:**
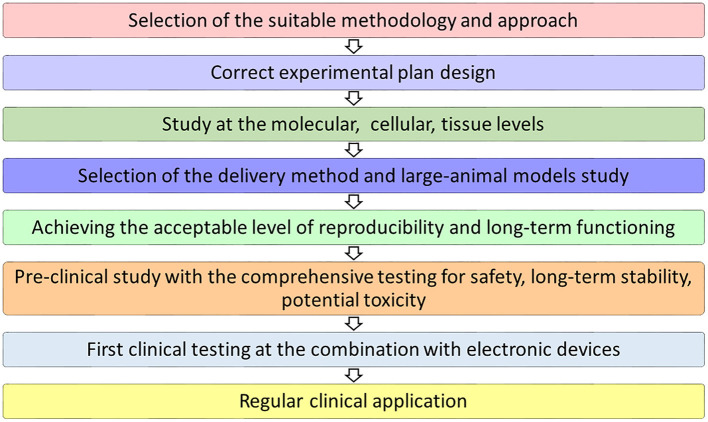
Milestones for the clinical translation of a bioengineered pacemaker.

In the path toward natural rhythm restoration (Figure 1), several technological advances are deeply contributing to an increased understanding of conductance (patho)physiology (Franco and Icardo, 2001; Goodyer et al., 2019; van Eif et al., 2020; Padala et al., 2021), as well as to its more feasible addressing for resolution. Methodologies based on naturally pacing cells, stem cell differentiation, gene engineering, tissue engineering, and/or their combination have been rehearsed so far *in vitro* and *in vivo* with varying success rates in restoring adequate cardiac pacing and conduction.

### Cell-Based Approaches Using SAN Cells

After the initial unsuccessful, autologous, allogeneic, and xenogeneic transplantation trials of the SAN tissue (Rylant, 1927; Ernst and Paulson, 1962; Starzl et al., 1963; Morishita et al., 1981), subsequent attempts were realized using the methodologies characterized by an inferior technical demand.

One of the first approaches utilized to re-establish the biological pacemaker function was the cell-based one, relying on the concept of transplanting not a complete SAN tissue, but only its cells, possibly mixed with atrial cardiomyocytes. Typically, these methodologies are focused on the intrinsically electrogenic ability of the injected cells, which are expected to act as an ectopic pacemaker after functional electrical coupling with host working cardiomyocytes (Table 1).

**Table 1:** Cell-based biopacemaking approaches using SAN cells.

**SAN cells**	**Methodology and experimental details**	**References**
Canine, mixed atrial cardiomyocytes	Dissociated fetal canine atrial cardiomyocytes were transplanted into the left ventricle of an adult dog model of X-linked muscular dystrophy. Electrical and mechanical coupling between the host and donor cardiomyocytes were observed.	Ruhparwar et al., 2002
Human, mixed atrial cardiomyocytes	Dissociated human atrial cardiomyocytes containing SAN PCs were injected into porcine left ventricles. Functional junctions, effective pacing, and optimal autonomic reaction between the donor cells and host cardiomyocytes were identified.	Lin et al., 2005
Canine SAN cells	SAN cells were injected into the right ventricle subepicardial free wall and dogs were monitored for 2 weeks. Pacemaker function was assessed by overdrive pacing and IV epinephrine challenge. SAN cells expressed a time-dependent inward current (If) activating on hyperpolarization. Brisk catecholamine responsiveness occurred. However, dogs implanted with autologous SAN cells manifested biological pacing properties dissimilar from those of the anatomic tissue, thus evidencing a correlation between the substrate environment and phenotype modification in injected cells.	Zhang et al., 2011

*SAN, sinoatrial node; PCs, pacemaker cells*.

Ruhparwar et al. (2002) dissociated the fetal canine atrial cardiomyocytes and injected them into the left ventricle of an adult dog model of X-linked muscular dystrophy. Successful electrical and mechanical coupling between host and donor cardiomyocytes was disclosed. Later, in 2005, the similar results were reported with a cell preparation of human atrial cardiomyocytes containing SAN PCs. When the mixed human atrial cells were injected into the porcine left ventricles, functional junctions, effective pacing, and an optimal autonomic response between donor cells and host cardiomyocytes were identified (Lin et al., 2005).

In another preclinical study, autologous SAN cells were injected into the myocardial wall of the right ventricle in a dog model after a complete heart block and the implantation of an electronic pacemaker. In spite of preserving the electrical activity, they showed different pacing features compared to their originating conduction system station. This observation highlights the relevant role of substrate conditioning in cell pacemaking activity (Zhang et al., 2011) and, hence, the still poorly studied but specific modulatory effect exerted by local cellular and extracellular microenvironments on the phenotype of exogenously introduced cells.

Although these initial cell-based strategies did not reach any clinical translation, they served as proof-of-concept for more advanced stem cell-based therapies and the next generation of pacemakers with optimal functioning after implantation in the selected regions of the heart.

### Cell-Based Approaches Using Pluripotent Stem Cell-Derived Cardiomyocytes

Pluripotent stem cells (PSCs), i.e., embryonic stem cells (ESCs) and induced PSCs (iPSCs), are considered the most promising stem cell types regarding their ability to differentiate into a virtually unlimited number of body cell types, including those derived from the cardiac lineage (Rajala et al., 2011; Kadota et al., 2020). An accurate methodology for cardiomyocyte differentiation and/or PC specification from human PSCs (hPSCs) is based on the features and patterns of the embryonic cardiac development and aims to recapitulate *in vitro* the stage-specific modulation of its signaling pathways. It includes experimental tactics of embryoid body development (Yang et al., 2008) or monolayer attachment culture with specific biochemical conditioning (Laflamme et al., 2007). Yet, the differentiation of PSCs to cardiomyocytes mainly results in a mixture of atrial, ventricular, and nodal cells; therefore, the ultimate goal to guide the differentiation into a desired subtype and to create the biological pacemaker appears challenging (Table 2) (Xu et al., 2002; He et al., 2003; Yang et al., 2008; Jung et al., 2012, 2014; Mandel et al., 2012; Müller et al., 2012; Christoforou et al., 2013; Birket et al., 2015; Protze et al., 2016; Chauveau et al., 2017; Dorn et al., 2018; Zhang and Huang, 2019; Zhang et al., 2019a; Yechikov et al., 2020).

**Table 2:** Cell-based biopacemaking approaches by PSC differentiation into cardiomyocytes.

**PSCs**	**Methodology and experimental details**	**References**
hESCs	Electrically active hESC-derived cardiomyocytes were transplanted into guinea pig hearts. Functional integration and pacing generation were achieved.	Xue et al., 2005
	Cardiomyocyte cell grafts were generated from hESC *in vitro* using the embryoid body differentiating system, this tissue formed structural and electromechanical connections with cultured rat cardiomyocytes. *In vivo* integration was shown in a large animal model of slow heart rate. The transplanted hESC-derived cardiomyocytes paced the hearts of swine with complete atrioventricular block.	Kehat et al., 2004
	hESC-derived cardiomyocytes were used to form scaffold-free patches (implanted on the epicardium) and micro-tissue particles (delivered by intramyocardial injection) into the ischemia/reperfusion injured athymic rat heart.	Gerbin et al., 2015
hiPSCs	SAN-like pacemaker cells from hiPSCs were identified as NKX2-5-negative, SIRPA-positive cardiomyocytes displaying pacemaker action potentials, ion current profiles, and chronotropic responses. When transplanted into the apex of rat hearts, SAN-like cells demonstrated pacemaking activity.	Protze et al., 2016
	hiPSC-derived cardiomyocytes were integrated into the host myocardium of AVN-blocked dogs and induced a biological pacemaking activity.	Chauveau et al., 2017

*PSCs, pluripotent stem cells; hESCs, human embryonic stem cells; hiPSCs, human induced pluripotent stem cells; SAN, sinoatrial node; AVN, atrioventricular node*.

Human ESCs (hESCs) are widely utilized in stem cell-based methodologies due to their ability to differentiate into spontaneously beating cardiomyocytes, which functionally express HCN channels (Kehat et al., 2001; Xu et al., 2002; He et al., 2003). For example, the functional integration and generation of the pacemaker activity were achieved in the transplantation experiments of electrically active hESC-derived cardiomyocytes into guinea pig hearts. Comprehensive optical mapping of the epicardial surface of these guinea pig hearts integrated with hESC-derived cardiomyocytes proved the extent of membrane depolarization being effective from the injection site to an adjacent myocardium as a bona fide sign of syncytium formation (Xue et al., 2005).

Indeed, human iPSCs (hiPSCs) are finding larger applications for the generation of PCs-like cardiomyocytes. Modern protocols currently allow to effectively generate hiPSCs-derived cardiomyocytes with the typical cardiac physiological features: a variety of ion channels, specific contracting apparatus, excitation, propagation, etc. (Xu et al., 2002; He et al., 2003; Yang et al., 2008; Jung et al., 2012; Mandel et al., 2012; Christoforou et al., 2013; Burridge et al., 2014; Birket et al., 2015; Dorn et al., 2018; Zhang and Huang, 2019). Moreover, biological pacing ability was demonstrated *in vitro* and *in vivo* (e.g., Protze et al., 2016; Chauveau et al., 2017).

Most approaches aiming at re-establishing the pacemaker activity relied on the injection of a heterogenous cardiomyocyte population derived from iPSC cardiac differentiation. For instance, after the delivery of iPSC-derived cardiomyocytes derived from embryoid body differentiation into AV block canine hearts in open thoracotomy, Chauveau et al. (2017) observed that these cells were integrated into the host heart tissue and were able to pace. However, this approach should be additionally optimized to increase the yield in the number of iPSC-derived PCs and reach a critical mass for effective and sustained pacing.

Notably, only a few studies attempted to overcome this issue by focusing on deriving SAN-like cells from the cardiac differentiation and pacemaker specification of hiPSCs (Müller et al., 2012; Birket et al., 2015; Protze et al., 2016; Zhang and Huang, 2019; Yechikov et al., 2020). Calcium-activated potassium channels were demonstrated in hPSCs as a valid target to generate an enriched population of PC-like cells by conditioning with 1-ethyl-2-benzimidazolinone (1-EBIO). After an initial induction of early mesodermal and cardiac genes (Brachyury, Isl1, and Myh6), conditioned cells displayed a small size, a high expression of the nodal marker HCN4, and a reduced content of myofibrils (Müller et al., 2012). Similarly, 1-EBIO might activate an atrial/PC cell differentiation program in conditioned ESCs and iPSCs. Birket et al. (2015) took advantage of lineage tracing to isolate the population of PCs from NKX2.5^eGFP/w^ hESCs. Upon cardiac differentiation through a doxycycline-inducible MYC transgene and/or fibroblast growth factor (FGF)/bone morphogenetic proteins (BMP) signaling modulation, they observed that eGFP-negative cells were expectedly NKX2.5-negative but also expressed ISL1 and the posterior heart field marker podoplanin (PDPN) at high levels. This pattern of expression in these cardiac progenitors is typical of the native SAN during development. After further differentiation, these cells also showed a strong induction of TBX3, SHOX2, TBX18, and HCN4 and displayed the electrophysiological features of PCs. So-selected eGFP-negative, ISL1-positive, and NKX2.5-negative cardiac progenitors were demonstrated to be clonogenic and multipotent (Birket et al., 2015), therefore opening the way to further studies on development and disease modeling. However, the reliance of this PC generating system on a MYC transgene moves away from the clinical path and may require alternative strategies for a regenerative medicine application in humans.

A promising transgene-independent method for generating pure SAN-like PCs from hiPSCs was proposed by Protze et al. (2016) by a stage-specific manipulation of developmental signaling pathways. In these experiments, iPSC cardiac differentiation and PC specification were induced by time-controlled administration of several transcription factors, as first BMP4, Activin A, and bFGF, and, hence, the inhibitors of Wnt production 2 (IWP2) and vascular endothelial growth factor (VEGF), in order to induce cardiac mesoderm and cardiomyocyte generation. SAN-like PCs were selected in the whole population as NKX2.5-negative, SIRPA-positive cardiomyocytes expressing the typical SAN lineage markers (TBX18, SHOX2, TBX3, and ISL1), classic ion current profiles (e.g., I_f_ and I_KAch_), chronotropic responses, and the ability to fire pacemaker action potentials and pace ventricular cardiomyocytes *in vitro*. After the transfer into the apex of rat hearts submitted to AV block, these SAN-like cardiomyocytes demonstrated pacing activity in the host tissue and were able to function as a biological pacemaker (Protze et al., 2016).

Zhang et al. (2019b) disclosed the outcomes of a double-reporter system based on TBX5^Clover2^ and NKX2.5^TagRFP^ developed with CRISPR-Cas9 technology to isolate TBX5-positive, NKX2.5-negative cardiac progenitors from the differentiated hiPSCs. These progenitors were identified as epicardial, by expressing the markers WT1 and TBX18, and they were found also positive for SHOX2, TBX3, HCN1, HCN4, and KCNJ3. PC-like cells differentiated from these progenitors showed a typical action potential morphology (in 80% of the total cells) and distinctive parameters (Zhang et al., 2019a). Unfortunately, this characterization did not identify a specific surface marker, which could be suitable to enrich this TBX5-positive, NKX2.5-negative subset of progenitors independently from the reporter system.

More recently, Yechikov et al. (2020) attempted to investigate the PC-like specification through Nodal inhibition. In the development of cardiac mesoderm, the inhibition of Nodal signaling downregulates a transcription factor, PITX2c, which represses SAN formation in the left atrium. Based on this rationale, iPSC-cardiomyocytes were submitted to Nodal signaling repression by the specific inhibitor SB431542. This conditioning actually induced the generation of a mixed population, also with nodal-like electrophysiological characteristics and higher expression of the transcription factors TBX3 and TBX18 (Yechikov et al., 2020), but not enriched or pure PCs.

Eventually, the identification of an effective specification protocol and/or a surface selection marker to enrich PC-like cells after PSC differentiation could anticipate the clinical application. As described previously, hPSC-cardiomyocytes, including enriched PC-like cells, were extensively investigated in various animal models (mouse, rat, guinea pig, pig, and primates) for the prospective of clinical transplantation (Kadota et al., 2020). In France, the phase I ESCORT trial by Menasché and colleagues evaluated the feasibility and safety to differentiate hESCs into ISL1-positive, CD15-positive cardiac progenitors for a clinical-grade therapeutic approach for the severe dysfunction of the left ventricle. No teratomas or arrhythmias were observed in the median of 18 months (Menasché et al., 2018). The first trial based on human allogeneic iPSCs started in Japan in 2019 to study the short-term efficacy of severe ischemic cardiomyopathy treatment by combining the derived cardiac progenitors and cell sheet technology (ClinicalTrials.gov Identifier: NCT04696328). As such, other clinical applications of PSC-derived cardiomyocytes are expected, including biopacemaking. However, challenging aspects for restoring pacing—apart from the relative pureness of hiPS-derived PCs—remain the delivery modality and cell injection substrate. Different approaches of hPSC-cardiomyocytes transplantation into the recipient cardiac tissue are currently tested (e.g., direct intramuscular injection or cell sheet epicardial patch techniques), which were often proved to allow for the formation of gap junctions between added cells and recipient tissues, and electrical coupling (Zhang et al., 2001; Hamdi et al., 2011; Kawamura et al., 2013; Narita et al., 2013; Tano et al., 2014; Gerbin et al., 2015; Kadota et al., 2020). It is still to be confirmed whether these methods could be equally suitable to guarantee adequate and functional integration also for hPSC-derived PC-like cells.

### Gene-Based Approaches

Gene delivery was long time studied for the potential application in biological pacemaker regeneration methodologies (Table 3). It aims at overexpressing a gene codifying for an ion channel or another protein relevant in PC electrophysiology by adopting viral or non-viral transfer strategies.

**Table 3:** Gene-based biopacemaking approaches.

**Genetic engineering**	**Methodology and experimental details**	**References**
Dominant-negative inhibition of Kir2-encoded inward-rectifier potassium channels	*In vivo* viral gene transfer to transform quiescent heart-muscle cells into PCs was performed. After the construct injection into the left ventricular cavity of guinea pigs, successful generation of spontaneous, rhythmic electrical activity in the ventricle was achieved.	Miake et al., 2002
Human β2-adrenergic receptor transfection	The effects of β2-adrenergic receptor transfer were studied: *in vitro* (murine embryonic cardiac myocytes transient transfection with plasmid constructs), *ex vivo* (murine neonatal cardiac transplantation model), and *in vivo* (injection into the right atrium of the endogenous heart).	Edelberg et al., 1998
	Plasmids encoding human β2-adrenergic receptor were injected into the right atria of native Yorkshire pig hearts. A significant increase of chronotropy compared with control injections was achieved.	Edelberg, 2001
HCN1 gene overexpression	HCN1 mutant (three deleted residues: HCN1-AAA) showed activation kinetics similar to SAN and induced pacing activity in porcine models.	Tse et al., 2006
HCN2 gene overexpression	HCN2 gene overexpression increased the heart rate and generated biological pacemaker activity in canine models.	Qu et al., 2003
Dual gene constructs HCN2/SkM1	Dual gene constructs HCN2/SkM1 were transduced into the left bundle branches in the models of complete AVN block dogs. Complete restoration of the heart rate was demonstrated.	Boink et al., 2013
Adenylate cyclase type VI (AC-VI) overexpression	Adenoviral gene transfer of AC-VI induced pacemaker activity in the AVN block model in adult pigs.	Ruhparwar et al., 2010
Adenoviral vector cocktail (K(AAA) + H2), expressing Kir2.1AAA and HCN2 genes	An adenoviral vector cocktail (K(AAA) + H2), expressing Kir2.1AAA and HCN2 genes, was injected into the AV junctional region in a model of AV block in pigs.	Cingolani et al., 2012
AC1 or HCN2/AC1 overexpression	Ca^2+^-stimulated adenylyl cyclase AC1 or HCN2/AC1 overexpression in left bundle branches provides highly efficient biological pacing and greater sensitivity to autonomic modulation than HCN2 alone.	Boink et al., 2012b

*PCs, pacemaker cells; SAN, sinoatrial node; AVN, atrioventricular node*.

*De facto*, the first effective gene-based approach for the generation of a biological pacemaker was applied by Miake et al. (2002) by using the viral gene transfer to transform quiescent heart-muscle cells into PCs. This study was focused on the inhibition of the endogenous inward rectifier potassium current (I_K1_) to prevent the automaticity suppression in guinea pig ventricular myocytes. Reduction in the number of inward rectifier potassium ion channels (encoded by the KIR2 gene family; KCNJ2) in the myocardium by overexpressing a KIR2.1-dominant-negative construct (KIR2.1AAA) was adopted. The suppression of I_K1_ induced ventricular cardiomyocytes to depolarize spontaneously, thus producing pacemaker activity (Miake et al., 2002). Further research focused on the KIR2.1AAA-based approach indicated that the overexpression of KIR2.1AAA not only prompts spontaneous membrane depolarization but also additionally triggers the action potential prolongation in case of lower mutant gene overexpression (Miake et al., 2003). However, there are some limitations regarding the use of Kir2.1-induced suppression as it could be associated with the heterogeneous expression of the ion channel between transduced and non-transduced regions, and, hence, arrhythmogenic electrical instability could be triggered (Miake et al., 2003; Sekar et al., 2009).

A growing body of evidence supports the hypothesis that the upregulation of exogenous β2-adrenergic receptors in the right atrium might cause an increase in the heart rate (Edelberg et al., 1998; Edelberg, 2001; Greene et al., 2012). Experimental data demonstrated that upon the atrial injection of the β2-adrenergic receptor construct, cardiac chronotropy enhanced up to 40% in mice (Edelberg et al., 1998) and up to 50% in pigs (Edelberg, 2001). In fact, this targeted, non-viral overexpression of β2-adrenergic receptors increases the protein availability for binding to endogenous catecholamines. Additional research confirmed that β2-adrenergic receptors colocalize with some ion channels, which are crucial for the correct cardiac function and heart rate. It was also established that β2-adrenergic receptors could create protein complexes with the pacemaker HCN4 channel and other subtypes of HCN channels (Greene et al., 2012). Nevertheless, this approach has only a chronotropic effect and does not increase the number of pacemaker channels. Moreover, uncertainties associated with the duration of construct expression might impede β2-adrenergic receptor overexpression from being effectively translated in the clinics.

A locally enhanced chronotropic activity might potentially be achieved through the HCN overexpression in the subsidiary atrial pacemaker tissue, which is physiologically bradycardic but shares several characteristics with SAN, including the differential expression of TBX3, HCN1, Nav1.5, and Cx43 with respect to the right atrium. It was shown, in fact, that pacing could be accelerated by the localized HCN2 or HCN212 overexpression (Morris et al., 2013), hence, advancing proof-of-concept for the clinical use of this subsidiary atrial pacemaker tissue for biopacemaking in the treatment of sick sinus syndrome. Translational interest was also dedicated to unravel the molecular basis of the link between the HCN repression and decreased heart rate. D'Souza et al. (2017) first demonstrated a prominent role for a microRNA, i.e., miR-423-5p, in the downregulation of HCN4 during bradycardia. Such a finding could explain the reduced heart rate observed in athletes and, ultimately, the sinus nodal dysfunction often diagnosed in elders who played sports at competitive levels. Yanni et al. (2020) similarly evidenced in models of heart failure with sinus bradycardia that the downregulation of the pacemaker ion channel HCN4 and its corresponding ionic current I_f_ is associated with the upregulation of another microRNA (miR-370-3p). Thus, the regulation of these specific miRs deserves more attention for possible pharmacological targeting in therapeutic strategies preventing the irreversible dysfunction of SAN.

Due to the extreme relevance of I_f_ current on pacemaking, additional gene-based approaches for the bioengineering of biological pacemakers are focused on the local transfer of a unique HCN gene (Qu et al., 2003; Tse et al., 2006; Plotnikov et al., 2008; Boink et al., 2013). HCN2 gene overexpression can increase the heart rate as well as generate biological pacemaker activity, as first demonstrated by Qu et al. (2003) in a canine model. In this experiment, adenoviral HCN2 constructs were injected by open thoracotomy into the left atrial appendage. After suppressing sinus rhythm by vagal stimulation, a spontaneous rhythm was observed on day 4 after the injection (Qu et al., 2003). In dogs with a complete AV block, Bucchi et al. (2006) demonstrated that the biological pacemaker obtained by the gene transfer of mE324A, a mutant of murine HCN2 (mHCN2) genes, in the left bundle branch could function in tandem with electronic pacemakers, reducing the number of their beats, and conferring sympathetic responsiveness. Moreover, they showed that mE324A was more effective than mHCN2 in activating the pacemaker current and providing catecholamine sensitivity (Bucchi et al., 2006). In a porcine model of sick sinus syndrome (SAN radiofrequency ablation) supported by electronic pacemaker implantation, Tse et al. (2006) observed that the overexpression of an engineered HCN1 construct through a somatic gene transfer could restore a physiological heart rate and reliable pacing of the myocardium by reducing the need for electronic pacing.

Shortcomings associated with the sole HCN-based genetic engineering for the biological pacing generation are related to relatively low autonomic sensitivity and can be overcome by dual-gene overexpression strategies with the skeletal muscle Na^+^ channel (SkM1) or adenylyl cyclase (AC) genes (Boink et al., 2012b, 2013). Adenoviral dual gene construct HCN2/SkM1 transduction into left bundle branches was demonstrated to restore the heart rate in complete AV block dogs. It was proved that upon the local overexpression of HCN2 and SkM1, no dependency on the electronic reserved pacing as well as better autonomic responsiveness were established (Boink et al., 2013). *In vivo* adenoviral gene transfer of AC type VI was demonstrated to induce pacemaker activity in an AVN block model in adult pigs (Ruhparwar et al., 2010). However, this rhythm was initiated only after isoprenalin administration and, thus, limiting the suitability of this approach only to preclinical study. Another AC overexpression-based modality using AC1 gene in combination with HCN2 revealed superior biological pacing and a higher degree in autonomic modulation than HCN2 alone (Boink et al., 2012b).

A different dual delivery strategy was applied by Piron et al. (2008) that, through a non-viral system (poloxamines), overexpressed HCN2 and β2-adrenergic receptor genes in the ventricular myocardium of a mouse model of AV block. Functional pacemaking and chronotropic regulation were achieved for a relatively long experimental period (Piron et al., 2008).

### Combined Gene-Cell Approaches

Gene-cell combinations explore the transfer of cells together with pacemaker genes into the heart to generate biopacemaking. The cells act, therefore, as delivery platforms for PC ion channels (Table 4).

**Table 4:** Combined gene-cell biopacemaking approaches.

**Gene-cell approach**	**Methodology and experimental details**	**References**
HCN1-expressing fibroblasts fused with freshly isolated myocytes	HCN1-expressing fibroblasts were fused with freshly isolated guinea pig ventricular myocytes and formed fibroblast-myocyte heterokaryons with biological pacemaker activity.	Cho et al., 2007
HCN1-expressing hMSCs	Genetically-engineered MSCs transfected with the human HCN1 gene expressed pacemaker I_f_ current. The effect of the hHCN1-transfected MSCs on cardiomyocyte excitability was determined by coculturing genetically engineered MSCs with neonatal rabbit ventricular myocytes.	Zhou et al., 2013
HCN2-expressing hMSCs	Genetically modified hMSCs expressed functional cardiac pacemaker gene HCN2 and induced spontaneous pacemaker activity triggering the contraction of ventricle cardiomyocytes *in vitro* and *in vivo*.	Potapova et al., 2004
	HCN2-expressing hMSCs were introduced into the right ventricular apex in dogs and biological pacemaker activity was obtained. Pacing was stable for 6 weeks with no cellular or humoral rejection.	Plotnikov et al., 2007
HCN4-expressing rabbit MSCs	*In vivo* integration and pacing function were achieved after the transplantation of mHCN4-modified rabbit MSCs into the rabbit left ventricle free wall epicardium. Pacing function of the modified MSCs persisted for at least 4 weeks after transplantation.	Zhang et al., 2012
HCN4-expressing rat MSCs	Genetically modified rat MSCs carrying HCN channels expressed pacemaker I_f_ current *in vitro*. Pacemaking activity was observed after transplantation into the rat host heart.	Nong et al., 2013
TBX18-expressing cardiomyocytes	The conversion of rodent cardiomyocytes to SAN cells *in vitro* and *in vivo* using the expression of Tbx18 was performed. Focal Tbx18 gene transfer in the guinea-pig ventricle induced ectopic pacemaker activity, correcting a bradycardic disease phenotype.	Kapoor et al., 2013
SHOX2-overexpression in ESCs	The overexpression of SHOX2 induced the differentiation of ESCs into pacemaker cells and the transplantation of embryoid bodies from SHOX2-transduced ESCs into the left ventricles of rat hearts with a complete heart block. Consistent pacing ability was demonstrated.	Ionta et al., 2015
HCN2/SkM1-overexpression in CPCs	Functional delivery of HCN2/SkM1 *via* human CPCs demonstrated effectiveness in bradycardia models. In particular, the lentiviral transduction of HCN2 and SkM1 was more efficient than their nucleofection-mediated gene transfer. Moreover, virally transduced cells survived better *in vivo*.	Végh et al., 2021

*MSCs, mesenchymal stem cells; hMSCs, human MSCs; SAN, sinoatrial node; ESCs, embryonic stem cells; CPCs, cardiomyocyte progenitor cells*.

Pioneering studies on this approach were advanced in 2007: their experimental concept was based on the chemically induced fusion of cardiomyocytes and syngeneic fibroblasts, which had been manipulated to express HCN1 pacemaker channels. HCN1-expressing fibroblasts were conditioned to fuse with freshly isolated guinea pig ventricular myocytes to form fibroblast-myocyte heterokaryons displaying biological pacemaker activity (Cho et al., 2007).

Apart from cell fusion, another system to deliver pacemaker genes in the heart revealed its potential: genetically modified human mesenchymal stem cells (hMSCs) were proved to express functional cardiac pacemaker HCN2 channels and induce spontaneous pacemaker activity, triggering the contraction of ventricle cardiomyocytes *in vitro*, as well *in vivo* when injected into the sub-epicardial left ventricular wall (Potapova et al., 2004). With a similar approach, Boink et al. (2012a) effectively used canine mesenchymal stem cells (cMSCs) to deliver SkM1 channels and their derived current into the injected epicardial border zones. Normal conduction and no arrhythmias were achieved by this cell-mediated delivery. As a further proof of the efficacy of SkM1-based approaches, functional delivery of HCN2/SkM1 *via* another platform, namely human cardiomyocyte progenitor cells (CPCs), was recently demonstrated in bradycardia models. In particular, the lentiviral transduction of HCN2 and SkM1 was more efficient than their nucleofection-mediated gene transfer. Moreover, virally transduced cells survived better *in vivo* (Végh et al., 2021). These notable pieces of evidence are supportive of the high translational potential of this gene delivery modality in the treatment of SAN bradycardic pathologies. Nevertheless, the use of lentiviral vectors is not free from safety issues due to the tumorigenic risks associated with random insertion in the cell genome.

In other animal studies based on hMSCs transduced with HCN1, HCN2, or HCN4, the ability of electrical coupling with cardiomyocytes and the generation of I_f_ current were also confirmed (Zhang et al., 2012; Nong et al., 2013; Zhou et al., 2013). As recently reviewed by Pittenger et al. (2019), hMSCs have aroused an incredible translational interest in many cell therapy applications due to their peculiar repertoire of major histocompatibility complex (MHC) molecules, anti-inflammatory paracrine effect, and ability to suppress mixed lymphocyte reactions, which render them immunotolerated in allogeneic donations, too. However, some experimental and clinical complications could be related to the possible migration of MSCs from injection sites and their differentiation into other cardiomyocytes or cardiac cells (Quinn and Flake, 2008; Li, 2012). Such a type of occurrence could cause a gradual, time-dependent loss of pacing. Further research should focus on biotechnological modalities to encapsulate MSCs and/or anchor cell clusters for the site-specific delivery of HCN2 and SkM1 ion channels. In all the cases, permanent and stable coupling between HCN-transduced MSCs and the working myocardium should be achieved for further clinical applications (Boheler, 2004; Li, 2012). These issues could be solved by using other cellular delivery platforms that can guarantee the sustained pacemaker function. As an example, the aforementioned human CPCs are an endogenous cell population of the heart and showed the survival and functional integration in the injected peri-ischemic cardiac sites in a long-term follow-up (Smits et al., 2009), thus being a valid alternative to hMSCs for biopacemaker gene delivery (Végh et al., 2019, 2021).

### Transcription Factor-Based Reprogramming Approaches

An advanced approach for pacemaker bioengineering is based on transcription factor manipulations and is currently extensively studied with a view to clinical translation. The transfer of genes encoding different fundamental transcription factors in conduction system development could have the prospective to generate faithful replicas of actual PCs (Table 5).

**Table 5:** Reprogramming biopacemaking approaches.

**Reprogramming approach**	**Methodology and experimental details**	**References**
TBX3-induced reprogramming	TBX3 overexpression in rat cardiomyocytes *in vivo* induced the reduction of intercellular coupling and I_K1_ density, but failed to establish ectopic pacemaking and I_f_ current.	Bakker et al., 2012
TBX18-induced reprogramming	The conversion of rodent cardiomyocytes to SAN cells *in vitro* and *in vivo* by the expression of Tbx18 was performed. Focal Tbx18 gene transfer in the guinea pig ventricle induced ectopic pacemaker activity, correcting a bradycardic disease phenotype.	Kapoor et al., 2013
	TBX18 gene transfer created biological pacemaker activity *in vivo* in a complete heart block in pigs.	Hu et al., 2014
	TBX18 gene delivery resulted in antegrade conduction rescue in a preclinical model of right ventricular pacing-induced cardiomyopathy.	Dawkins et al., 2019

*SAN, sinoatrial node*.

Even though directed cardiomyogenesis from fibroblasts was significantly enhanced in the last few years by reprogramming with several cardiopoietic transcription factor combinations (Ieda et al., 2010; Inagawa et al., 2012; Protze et al., 2012; Qian et al., 2012; Christoforou et al., 2013), targeted differentiation of fibroblasts or working cardiomyocytes into PCs, particularly SAN and AVN cells, remains to be poorly studied. It is known that PC development and differentiation are significantly modulated by specific transcriptional regulators, among them SHOX2, TBX3, TBX5, and TBX18 (Blaschke et al., 2007; Christoffels et al., 2010; Munshi, 2012; Cho, 2015; Gorabi et al., 2019a,b; Raghunathan et al., 2020; van Eif et al., 2020).

Hoogaars et al. (2007) performed a fundamental study on the role of the transcriptional repressor TBX3 in the development of the cardiac conduction system. TBX3 expression defines the SAN region, which initiates a distinct gene expression program compared to the adjacent atrial cells. Lineage segregation of TBX3-negative atrial and TBX3-positive SAN precursor cells was observed after atrial gene program initiation. TBX3-dependent SAN specification and formation as well as the regulation of the pacemaker gene expression program were proved. Later, Bakker et al. (2012) established the effect of TBX3 on the adult heart in the context of its ability to reprogram terminally differentiated working cardiomyocytes into PCs: reduced intercellular coupling and I_K1_ density, but no ectopic pacemaking and I_f_ current were revealed. The pro-pacemaking ability of the transcriptional factor TBX3 was also tested by its *in vitro* overexpression in ESCs. Forward specification of PSCs with the nodal cell inducer TBX3 was used in combination with Myh6-promoter-based antibiotic selection as a successful strategy to increase PC enrichment. In fact, 80% of the selected cells exhibited nodal-like electrophysiological characteristics, enhanced HCN4 levels, and fired spontaneous action potentials (Jung et al., 2014).

Embryonic T-box transcription factor TBX18 is another crucial PC transcriptional factor: it is necessary to develop the SAN head (right caval vein myocardium) and induce the differentiation of SAN myocardium (Wiese et al., 2009). The first successful realization of a genetic engineering methodology based on this transcriptional factor was introduced in 2013 and 2014 by overexpressing the gene encoding the human TBX18 to induce the conversion of adult ventricular cardiomyocytes into SAN-like cells (Kapoor et al., 2013; Hu et al., 2014). The latter displayed various phenotypic and functional characteristics of the native PCs. They not only initiated a biological pacemaker rhythm from the site of injection but also were shown to be sensitive to catecholamines (Kapoor et al., 2013). In other experiments, TBX18-expressing adenoviruses were delivered into the interventricular septa of pigs with a complete heart block: TBX18 overexpression enhanced the heart rate and also resulted in automaticity originating from the focal site of gene injection (Hu et al., 2014). However, biopacemaking was maintained as sustained for no more than 2 weeks due to the reasons that were not investigated but hypothesized as related to inflammation, immune reactions, or unintended reprogramming of AVN cells. These studies were only the pioneering ones in the biological pacing generation through TBX18 reprogramming, and, currently, the attempts for the successful realization of this methodology continue. TBX18 gene-based reprogramming into PC-like cells was demonstrated *in vitro* starting from neonatal rat fibroblasts, vascular smooth muscle cells from ascending aorta, and adipose tissue MSCs (Yang et al., 2016; Quan and Huang, 2018; Wang et al., 2019). Experiments with TBX18 gene overexpression in hiPSC-cardiomyocytes to induce PC-like cells demonstrated the effectiveness of this approach independently from the gene delivery modality (Gorabi et al., 2019b). The essential role of TBX18 was also demonstrated recently by Dawkins et al. (2019) in a preclinical model of right ventricular pacing-induced cardiomyopathy. It was shown that it is possible to prevent and reverse cardiomyopathy signs by the strategy of antegrade conduction rescue *via* TBX18 biological pacing. Despite the promise generated by TBX18 gene delivery for pacemaking restoring, several concerns for its safe clinical application remain in addition to the aforementioned unsolved issue of temporary effect. Whenever a permanent overexpression will be reached, the risk of interference with epithelial-mesenchymal transition can be hypothesized depending on the gene delivery site. Such a consequence cannot be excluded because TBX18, together with Wilms' tumor homolog 1 (WT1), is upregulated in the developing epicardium and injury-activated epicardial stem cells, giving rise, through the epithelial-mesenchymal transition, to the fibroblasts and smooth muscle cells of the coronary arteries (Takeichi et al., 2013) and/or cardiomyocytes (Smart et al., 2011).

The ability of the transcriptional factor SHOX2 to activate PC differentiation was tested *in vitro* by the adenoviral transfer of human SHOX2 into mouse ESCs. The transplantation of embryoid bodies from SHOX2-transduced ESCs into the left ventricles of rat hearts with a complete heart block demonstrated the consistent pacing ability nascent from the injection sites, as recorded by *ex vivo* optimal mapping 2–4 days after injection (Ionta et al., 2015). Despite the short window of evaluation, SHOX2-transduced embryoid bodies proved to immediately integrate with the host ventricular myocardium. Notably, neither SHOX2 gene delivery nor mouse embryoid bodies appeared to trigger an immune response in this discordant, immunocompetent transplantation model. Longer follow-up of these SHOX2-transduced embryoid bodies in the rat heart as well as direct *in vivo* reprogramming using SHOX2 adenoviral construct could shed more light on possible safety complications.

ISL1 transcriptional factor was also studied for its ability to initiate the PC differentiation: ISL1 overexpression in ESCs and in *Xenopus laevis* embryos stimulates the increase of the cardiomyocyte precursors' differentiation toward prevalently nodal cells. Enhanced HCN4 expression and increased cellular automaticity were also observed (Dorn et al., 2015). These outcomes confirmed in two systems are suggestive for the potential use of ISL1 as a pro-pacemaking transcriptional factor *in vivo*. Although no animal investigation has been performed yet, another *in vitro* study on adipose tissue-derived MSCs demonstrated the obtainment of PC-like cells through lentiviral delivery of ISL1 combined with TBX18. Transfected cells expressed TBX3, HCN4, cTnT, and Cx45; moreover, they possessed a functional I_f_ current (Zhang and Huang, 2019). Even if the used lentiviral gene expression system opens up many safety concerns, the fact that ISL1/TBX18 double gene transfer may be effective in initiating the SAN program also on mesodermal (stem) cells can be seen as further evidence of the validity of this approach in somatic cell reprogramming *in vivo*.

As it can be noted from these studies, investigation on a multifactorial gene strategy for direct reprogramming of somatic cells into PCs with transcription factors is still missing. Nam et al. (2014) played on the combination of transcription factors demonstrated efficacious for cardiomyocyte induction (Ieda et al., 2010; Protze et al., 2012; Qian et al., 2012). By adopting a highly reliable HCN-GFP reporter for PC tracking, they observed that the genetic cocktail of the selected cardiogenic transcription factors, including GATA4, HAND2, MEF2c, and TBX5, induced nearly 30% of bona fide PCs in transgenic fibroblasts without passing through a cardiac progenitor intermediate (Nam et al., 2014). Obviously, such a transcriptional gene transfer system cannot be clinically translated for a reliable biopacemaking as 70% of the induced cardiomyocytes were atrial or ventricular. However, the *in vitro* mechanistic platform developed in this study might possess strong prediction ability in identifying other combinations of transcription factors more effective in SAN program reactivation.

The reprogramming of somatic cells, such as cardiac fibroblasts, atrial, and ventricular cardiomyocytes, into functional hiPSC-pacemaking cardiomyocytes with adequate PC-like electrophysiological activity using various transcriptional factors might be likely the most straightforward approach for human cell-based biopacemaker engineering. Nevertheless, further studies are still required to find the suitable gene cocktails able to specifically initiate SAN program *in vitro*, test their validity, long-term efficacy, as well as possible concerns *in vivo*. In addition, careful attention should also be addressed in preclinical studies to evaluate improved and minimally invasive delivery modalities to be translated into the clinics with ease and safety.

### Tissue Engineering and Biohybrid Approaches

The multidisciplinary field of tissue engineering and regenerative medicine revealed its potentiality in many cardiovascular applications, such as the generation of heart valve replacements and cardiac tissue constructs (e.g., Iop et al., 2009, 2014, 2018; Dal Sasso et al., 2020; Zouhair et al., 2020). Tissue engineering is increasingly utilized for the development of biopacemakers (Table 6). Due to the architectural and functional complexity of nodal tissues, such a strategy might fully regenerate cardiac conduction by combining scaffolds and cells opportunely in biomimetic, bioequivalent tissue constructs. However, the drawbacks of the first SAN tissue transplantation experiments (Starzl et al., 1963; Morishita et al., 1981) as core necrosis, immune response, and non-integration might hamper the success of tissue engineering approaches and have to be excluded to prevent any condition leading to graft failure.

**Table 6:** Tissue engineering and hybrid biopacemaking approaches.

**Tissue engineering**	**Methodology and experimental details**	**References**
Tissue engineered cardiac pacemakers	A liquid mixture of skeletal myoblasts, collagen, and Matrigel was cast into molds, allowed to solidify, and statically cultured before being implanted into the cardiac atrioventricular groove of syngeneic rats. In 30% of the treated animals, the pacing was established.	Choi et al., 2006
	Cardiac and endothelial progenitors were opportunely mixed with Matrigel to obtain vascularized tissue-engineered pacemakers *in vitro*, which proved pacing activity in a rat model of SAN dysfunction.	Zhang et al., 2017
	A tissue-engineered construct composed of collagen sponges and cardiac progenitors derived from the human embryonic heart tubes was implanted in the rat atrioventricular groove. Around 60% of the implanted rats survived, and the pacing was maintained for about 3 months *in vivo*.	Zhang et al., 2019b
Biohybrid pacemaker devices	A biohybrid strategy combining smart coating biopolymers and cardiac electronic devices prevented the formation of fibrotic adherences *in vivo*.	Robotti et al., 2020

*SAN, sinoatrial node*.

A first tissue engineering effort to generate an AVN pacemaker was realized by Choi et al. (2006): a liquid mixture of skeletal myoblasts, collagen, and Matrigel was cast into molds, allowed to solidify, and statically cultured before being implanted into the cardiac atrioventricular groove of syngeneic rats. In 70% of the animals, AV conduction was not re-established. In the rest of the animals, it was permanently conveyed thanks to an implanted viable construct, promptly vascularized *in vivo*. This approach, however, is barely transferrable to human therapy for its low clinical grade (an xenogeneic material such as Matrigel) and reliance on non-specialized components (incompletely differentiated, non-cardiac cells, and immature, xenogeneic scaffold). Zhang et al. (2019b) reported the outcomes of the atrioventricular groove implantation of a tissue-engineered construct composed of collagen sponges and cardiac progenitors derived from the human embryonic heart tubes. Around 60% of the implanted rats survived, and the pacing was maintained for about 3 months *in vivo*. Again, such an approach could have difficult translation into the clinics depending on the ethical questions and legislative limitations on using human embryos issued by each country.

Cardiac organoid models were generated by inserting an iPSC-derived embryoid body into the engineered heart tissue (Schulze et al., 2019) by recapitulating the cell and extracellular matrix functional connections typically observed in the native nodal tissues.

The differentiation through the embryoid body method is possibly spontaneous and relatively easy to perform but might be associated with high yield variability in the achievement of cardiac progenitors with PC features and consequently to excessive system costs. A layer-by-layer cell coating technique has been proposed by Sasano et al. (2020) to more efficiently differentiate hiPSCs into a hierarchical cardiac tissue-mimetic structure.

Three-dimensional cardiac pacemaker spheroids were tissue-engineered by adopting somatic gene transfer technology of TBX18 (Grijalva et al., 2019). These tissue constructs spontaneously fired action potentials and induced cell contraction in co-cultured cardiomyocytes *in vitro*.

Vascularization is an important feature of a functional node. Cardiac pacemakers tissue-engineered with cardiac progenitors were promptly vascularized by adding *in vitro* endothelial progenitor cells to the cell component of the constructs realized with Matrigel and proved pacing activity in a rat model of SAN dysfunction (Zhang et al., 2017).

Through the STARS program BIOSAN, we also develop biological pacemakers based on the combination of natural scaffolds and induced PCs.

Alternatively, applying a biohybrid approach mixing smart polymers and electronic devices could respond to the immediate need to render an artificial pacemaker more natural-like for the body (Table 5; Feiner and Dvir, 2020). Such a strategy could prevent fibrotic reactions associated with the implantable device (Robotti et al., 2020), but it may not overcome other related limitations, as the lack of neuroautonomic responsiveness.

## Translational Challenges Toward the Clinical Application of Biological Pacemakers

Despite the variety of approaches that have been tested in different models, extensive and comprehensive additional studies are still necessary for further development and potential clinical application of biological pacemakers. For all current approaches, investigation on the optimization and the evaluation of safety, potential toxicity, long-term stability, and a variety of crucial parameters is definitely mandatory (Figure 1). Regarding recent studies and the obtained results, biological pacing therapy is much closer to the clinical application; however, numerous challenges still exist in this field (Rosen et al., 2011; Cingolani et al., 2018).

*In vitro* studies are fundamental to evaluate the proof-of-principle of novel biopacing concepts, but the ultimate efficacy test is on the animal model. Animal studies were performed with the primary goal to evaluate the efficacy, safety, stability, and other parameters of the different biological pacemaker approaches. Biological pacemaking was achieved in the majority of these models, which vary depending on the animal, duration of observation, delivery method, and time stability (Edelberg et al., 1998; Edelberg, 2001; Miake et al., 2002; Ruhparwar et al., 2002, 2010; Qu et al., 2003; Plotnikov et al., 2004, 2007, 2008; Potapova et al., 2004; Lin et al., 2005; Xue et al., 2005; Bucchi et al., 2006; Tse et al., 2006; Cai et al., 2007; Cho et al., 2007; Piron et al., 2008; Shlapakova et al., 2010; Zhang et al., 2011, 2012; Boink et al., 2012a,b; Cingolani et al., 2012; Kapoor et al., 2013; Morris et al., 2013; Nong et al., 2013; Hu et al., 2014; Ionta et al., 2015; Protze et al., 2016; Chauveau et al., 2017; Choudhury et al., 2018; Dawkins et al., 2019; Gorabi et al., 2019a; Végh et al., 2019, 2021).

Significant issues could prevent the successful adoption of the biopacing technologies in clinics: challenging delivery of genes and cells, inefficient integration and coupling, the risks of pro-arrhythmic effects of biological pacemakers, the possible teratogenic effects of stem cells or/and transcription factor-based approaches, and, not lastly, the ethical issues derived from the use of hESCs derivatives. One of the main concerns and potential limitations of biologic-driven automaticity is the possibility of ventricular arrhythmias and related life-threatening consequences.

Specific limitations might depend first on the delivery methods used to deploy genes, cells, or tissue-engineered constructs at the selected site. For all approaches pursued for biopacemaking, it is fundamental to find adequate delivery modalities that are easy to perform, minimally invasive, associated with low peri-intervention risks, and able to guarantee the sustained effect *in vivo*. Most gene or cell delivery methods are still invasive (open chest or thoracotomy), representing less attractive options for the future clinical application in humans (Miake et al., 2002; Ruhparwar et al., 2002, 2010; Qu et al., 2003; Potapova et al., 2004; Lin et al., 2005; Xue et al., 2005; Choi et al., 2006; Tse et al., 2006; Cai et al., 2007; Cho et al., 2007; Plotnikov et al., 2007; Boink et al., 2012a, 2013; Zhang et al., 2012; Kapoor et al., 2013; Nong et al., 2013; Ionta et al., 2015; Protze et al., 2016; Chauveau et al., 2017; Gorabi et al., 2019a). The development of minimally invasive, safer, and well-controlled delivery methods is a current research priority of biological pacemakers in the field of regenerative medicine. A catheter-based injection, especially when combined with fluoroscopic guiding, offered a safe and an effective option to deliver genes and cells with minimal invasiveness in preclinical biological pacing (Edelberg, 2001; Plotnikov et al., 2004, 2008; Bucchi et al., 2006; Cingolani et al., 2012; Hu et al., 2014). In particular, such a low invasive deployment modality for gene delivery causes minimum blood loss and pain and has a significantly lower risk of stroke due to the catheter insertion into the right-sided circulation. It is unquestionably a routine approach for many clinical applications and could be easily translated in humans for gene- and cell-based biopacemaking strategies, too.

Once the deployment has been performed, the initiation of biopacemaking is subordinate at first glance to the establishment of an immunotolerant state with respect to injected cells, genes, or tissue-engineered constructs. While MSCs are able to induce such a state (Pittenger et al., 2019), other cell types used as delivery platforms are considered immunogenic, as in the case of hESCs derivatives or SAN cell preparations from allogeneic donors (Drukker and Benvenisty, 2004). The adoption of iPSCs has allowed to partially skip this issue by employing patient-specific cells for reprogramming to pluripotency. PC-, atrial-, and ventricular-like cardiomyocytes differentiated from iPSCs were shown to display the same MHC repertoire as the somatic cells of the patient (Park et al., 2013). Although MHC-matched allogeneic iPSCs were recently reported to be well-tolerated in preclinical studies in immunocompetent animal models (Ishigaki et al., 2021), much concern related to several cases of rejection remains, leading to focus research on developing hypoimmunogenic iPSC lines (Deuse et al., 2019). Immunorejection might also happen with gene delivery, especially with viral constructs. The application of viral gene delivery strategies, particularly the ones depending on lentiviral vectors, might be more efficient than non-viral approaches to reach adequate overexpression of ion channels, other proteins essential to generate PC currents, and transcription factors relevant to start SAN program; however, the risks related to immunogenicity, but also random genome integration, and tumorigenicity cannot be underestimated at all. Some clinical trials with gene therapy, also combined with cell platforms, were stopped due to adverse events, as it was recently disclosed during the application of autologous hematopoietic stem cells transduced with a *BCL11A* mRNA-encoding lentiviral vector for the treatment of sickle cell disease (Esrick et al., 2021; Statement on NHLBI decision to pause the Pilot and Feasibility Study of Hematopoietic Stem Cell Gene Transfer for Sickle Cell Disease | NHLBI, NIH, 2021). As proposed by Végh et al. (2019), insertional mutagenesis could be prevented by adopting self-inactivating lentivirus or promoters with a weak-to-moderate activity. Non-viral strategies for gene delivery or reprogramming might be spare of some of these issues, but possible epigenetic modifications and genetic instabilities need to be investigated in depth, as they were observed also in hiPSCs reprogrammed with non-integrating methods (Schlaeger et al., 2015). Even if no data are available still regarding non-integrating gene delivery for *in vivo* biopacemaking, the strategies of microRNA-therapeutic silencing after myocardial infarction revealed to be immunotolerated in animal models (Liao et al., 2021). As far as concerning bioengineered conduction tissues, immunotolerance establishment depends both on scaffolds and cells, as well as on cell culture conditions (e.g., the use of xenogeneic reagents), employed to fabricate them; therefore, a careful selection of non-immunogenic components and/or manipulations for the antigenic moiety removal are required to prevent immune rejection, as in other cardiovascular tissue engineering applications (Iop et al., 2018).

Once biopacemaking has been activated, most of the gene- and cell-based approaches showed shortcomings in the mid-term maintenance of sustained performance. Such a limitation can be expected for tissue-engineered conduction bioequivalents, too. As mentioned before, yet a few biopacemaking studies demonstrated *in vivo* pacing for around 45 days while most of them reported function for no longerthan 2–4 weeks or did not assess pacing performance during longer follow-up (for instance, Plotnikov et al., 2007, 2008; Piron et al., 2008; Kapoor et al., 2013; Hu et al., 2014). Often, an increase in heart rhythm is reported after some days from the treatment but tends to decrease just as quickly. Some studies disclosed a loss of PC-like cells over time, as after transcription factor gene overexpression (Kapoor et al., 2013). Whether this time-limited efficiency is, due to late-onset immune reactions elicited against gene constructs or exogenous cells, transient gene expression (gene delivery), unintended cell targeting (gene delivery), reduced survival (cells), inability to integrate, core necrosis (tissue-engineered constructs), migration events, interference with other biological activities (e.g., epithelial-mesenchymal transition), conditioning from local microenvironment at the injection site (inflammatory state and/or region-specific electrophysiological characteristics), or a combination of these causes, it still remains to be fully elucidated.

Delivery constructs that target a specific cell type of the heart are not yet available, and vehiculated genes are, therefore, introduced at the site of injection where they are randomly inserted. It has been speculated that a non-targeted gene delivery might have a causative role in the unsustained activity at the AV level whether native, functional AVN PCs are the objects of the forced expression of channel proteins (Hu et al., 2014). Nanotechnology concepts applied in the cell targeting for cancer therapies (e.g., chimeric antigen receptor T-cell or CAR-T) (Ma et al., 2020) could be translated in the context of biopacemaking to induce the expression of the exogenous gene in a selected cell type, e.g., cardiac fibroblasts. Transient gene expression is related to the vector used to vehiculate the gene of interest. Lentiviral and retroviral delivery strategies are reliable for gene overexpression, also in the long term (Tse et al., 2006; Nong et al., 2013; Gorabi et al., 2019a; Végh et al., 2019, 2021), but might induce insertional mutagenesis as discussed before. The adenoviral transfer is non-integrating and efficient, but the expression is limited in time (Miake et al., 2002; Qu et al., 2003; Bucchi et al., 2006; Tse et al., 2006; Cho et al., 2007; Plotnikov et al., 2008; Ruhparwar et al., 2010; Cingolani et al., 2012; Kapoor et al., 2013; Hu et al., 2014; Ionta et al., 2015; Choudhury et al., 2018; Dawkins et al., 2019; Grijalva et al., 2019), and, thus, it cannot be considered as an option to be employed in the clinic for sustained biopacemaking. As advanced by Cingolani et al. (2018), this time-limited overexpression might, conversely, be ideal to establish temporary, hardware-free pacing in patients with device-related infections (bridge to device implantation), chronic atrial fibrillation, and congenital AVN block (*in utero*).

Inability to stably integrate, poor cell viability, and core necrosis phenomena might be a consequence of the lack of effective oxygen and nutrient supply, which is particularly problematic when transplanting thick tissues, as observed in the first trials with native SANs (Ernst and Paulson, 1962; Starzl et al., 1963; Morishita et al., 1981) and expected for tissue-engineered conduction constructs. Vascularization must be promptly established to prevent ischemic conditions and necrosis, also envisaging the *in vitro* creation of vessel networks in tissue engineering approaches (Zhang et al., 2017; Shah Mohammadi et al., 2020).

Another issue regarding the maintenance of biopacing is related to possible cell migration. Considering that the native conduction system possesses electrical insulation (Choi and Salama, 1998), all delivery strategies, including the ones intending to transplant tissue-engineered biopacemakers, should be developed considering this as one of the main prerequisites in order to prevent migration and dilution-like effects that could be possibly responsible for mid/long-term loss of function. Several strategies might be applied to avert migration. Anchoring modalities should be developed to maintain the cells at the site of injection. Encapsulation in biocompatible and semipermeable membranes could be advantageous, especially for cell-based approaches to favor viability and immunoisolation as already advanced clinically (Hardin-Young et al., 2000; Carlsson et al., 2018), as long as they do not prevent the creation of opportune electrical coupling for biopacemaking. Alternatively, for the strategies depending on cell delivery platforms, the use of cells belonging to the heart as cardiac stem cells might be resolutive (Végh et al., 2021). In conduction system tissue engineering, the combination with scaffolds characterized by slow biodegradation kinetics could be helpful. Mimicking or reproducing the natural electrical insulation of SAN and AVN should be pursued to prevent migration side effects as possible interferences with other biological functions and/or dedifferentiation/transdifferentiation.

Finally, suboptimal differentiation/specification and electromechanical coupling of the introduced or induced PC-like cells might be responsible for discontinuous pacing or the generation of arrhythmic foci (Plotnikov et al., 2008; Boink et al., 2012b; Shiba et al., 2014).

In order to deeply investigate these aspects, as well as safety and teratogenicity, accurate and long-term monitoring is essential. Typically, biological pacemaker activity and pro-arrhythmic effects were monitored by *in vivo* and *ex vivo* assessment. ECG, serial Holter monitoring, pacemaker log recordings, patch clamp, non-fluoroscopic magnetic electroanatomic system (CARTO), and optical mapping of the epicardial electrical activity in perfused, excided hearts are often employed to analyze biopacing activity (Miake et al., 2002, 2003; Ruhparwar et al., 2002, 2010; Qu et al., 2003; Xue et al., 2005; Choi et al., 2006; Tse et al., 2006; Plotnikov et al., 2007, 2008; Shlapakova et al., 2010; Zhang et al., 2012, 2017; Kapoor et al., 2013; Nong et al., 2013; Gorabi et al., 2019a). *In vivo* evaluations are more indicative of the induced biopacemaking activity because they allow to study electrical function in the living animal and assess the global function. More reliable results can be achieved by performing real-time, continuous ECG telemetry as it was done in the experiments with the model of complete heart block in pigs (Edelberg, 2001; Hu et al., 2014). Such continuous monitoring should be performed and extended for long-term follow-up in all large animal studies to evaluate any time-related dynamic change of the biological pacemaker. The most extended evaluation has been performed with a tissue-engineered biopacemaker (from 2 weeks to maximum 3 years), but unfortunately, no *in vivo* electrical assessment has been realized (Choi et al., 2006), which could have provided valuable information on the fate of the whole graft and its composing cells. Despite the abundance of animal studies, a comprehensive evaluation of safety, long-term stability, arrhythmogenicity, and toxicity remains still to be realized to date. Various *in vivo* dysfunctional rhythm models were successfully created to simulate, e.g., bradyarrhythmia, sick sinus syndrome, and AV block (Qu et al., 2003; Kehat et al., 2004; Plotnikov et al., 2004, 2007, 2008; Bucchi et al., 2006; Tse et al., 2006; Shlapakova et al., 2010; Zhang et al., 2012; Hu et al., 2014), by allowing to effectively test any one of the developed biopacemaker therapeutic concepts.

Another outstanding question in the clinical application of a biopacemaking therapy is related to ethics and costs. Most approaches so far explored rely on components, the in-human application of which might be estimated as controversial due to the derivation (e.g., ESCs and Matrigel) or associated health risks (viral delivery vectors, especially the integrating ones). With a considerable level of technology implemented in these approaches, the manufacturing costs are particularly high, representing a critical barrier for distribution in both industrialized and non-industrialized countries. As for other advanced therapeutic medicinal hypotheses, the balancing between benefits and risks associated to any biopacemaking approach must be critically valued in healthcare management programs to prevent any harmful exposure of patients affected by the disturbances of the conduction system. Indeed, the possibility of clinically testing these hypotheses depends on the local sanitary legislation, as well as on government/federal funding allocated, and, finally, on the consent of the patient. In the cases where no available treatment options are suitable to (re)establish rhythm functionality as for fetal subjects with congenital AV block or adult patients with device-related infections (Cingolani et al., 2018), compassionate use might eventually be authorized.

With the current level of knowledge and technology, a complete recovery of the physiological heart rate has not been achieved yet by using stem cell- and reprogramming-based approaches, but is confirmed at least temporarily using other methodologies as gene transfer. The delivery of genes codifying for essential proteins in PC electrical function or transcription factors initiating the conduction system program needs further optimization regarding the employed vectors and identification of effective cocktails. Cell-based approaches (mixed or pure SAN cell preparations or PC-like cells from the differentiated PSCs) and tissue engineering strategies will also require additional investigation before reaching the clinical application: the acceptable level of reproducibility and long-term functioning is not yet achieved. Although the biological pacing created by gene transfer showed hitherto to be the most successful biopacemaking strategy in short-term preclinical evaluations, the application of tissue engineering principles is expected to keep its promises to overcome all the limitations shown by the other approaches to clinically replace electronic pacemakers.

At the present moment, the best strategy for the first clinical testing of a biological pacemaker appears to be its combination with electronic devices (Plotnikov et al., 2004, 2008; Bucchi et al., 2006). Preliminary and first-stage biological pacing could be realized in the clinical conditions of permanent atrial fibrillation in combination with AV block or other complicated cases (Cingolani et al., 2018). Patients with sinus bradycardia could also be the candidates for the biological pacing with SAN transcription factor or microRNA (miRNA) reprogramming of atrial subsidiary pacemaker sites (Morris et al., 2013; D'Souza et al., 2017; Choudhury et al., 2018; Yanni et al., 2020).

## Conclusions

We overviewed a significant progress in the field of biological pacemaker development in the last few years. We have every reason to believe that modern biological pacing technology will demonstrate its validity in preclinical investigation *in vitro*, in long-term animal studies *in vivo*, and, finally, in the clinic. Various methodologies for biological pacemaker generation are being currently studied and show potentialities for further preclinical and clinical applications: cell-based, gene-based, combined gene-cell-based, transcription factor-induced reprogramming, and tissue engineering applications. Though all these approaches are focused on effective and safe biological pacing generation, they are founded on diverse strategies and delivery methods. In addition, limitations and crucial points for further clinical introduction (safety, long-term stability, and potential toxicity) differentiate them. Nevertheless, each of them potentially could be safe and effective and more promising compared to artificial electronic devices, so far clinically applied. In summary, biological pacemakers are expected to improve and expand the spectrum of the therapeutic strategies for the treatment of disorders in the cardiac conduction system.

## Author Contributions

LI conceived the manuscript. NN and LI contributed to the article and approved the submitted version.

## Conflict of Interest

The authors declare that the research was conducted in the absence of any commercial or financial relationships that could be construed as a potential conflict of interest.

## Publisher's Note

All claims expressed in this article are solely those of the authors and do not necessarily represent those of their affiliated organizations, or those of the publisher, the editors and the reviewers. Any product that may be evaluated in this article, or claim that may be made by its manufacturer, is not guaranteed or endorsed by the publisher.
